# OST-01, a natural product from *Baccharis coridifolia*, targets c-Myc-dependent ribogenesis in acute myeloid leukemia

**DOI:** 10.1038/s41375-024-02146-5

**Published:** 2024-01-17

**Authors:** HyunJun Kang, Dinh Hoa Hoang, Melissa Valerio, Khyatiben Pathak, Lianjun Zhang, Ralf Buettner, Fang Chen, Katrina Estrella, William Graff, Zhuo Li, Jun Xie, David Horne, Ya-Huei Kuo, Bin Zhang, Patrick Pirrotte, Le Xuan Truong Nguyen, Guido Marcucci

**Affiliations:** 1 Department of Hematologic Malignancies Translational Science, Beckman Research Institute and City of Hope National Medical Center, Duarte, CA USA; 2 Cancer & Cell Biology Division, Translational Genomics Research Institute, Phoenix, AZ USA; 3 Ostentus Therapeutics, Inc., Newport Coast, CA USA; 4 Beckman Research Institute, City of Hope National Medical Center, Duarte, CA USA; 5 Department of Molecular Medicine, City of Hope National Medical Center, Duarte, CA USA

**Keywords:** Acute myeloid leukaemia, Cell signalling

## To the Editor:

Treatment refractoriness and post-remission relapse in patients with acute myeloid leukemia (AML) are often due to the persistence of leukemia stem cells (LSCs) [1], suggesting more effective and less toxic therapies targeting LSCs are highly needed for AML patients. Natural products (NP) played a role in drug discovery [2, 3] and have been used in traditional medicine to treat a variety of conditions, including cancers [2, 4]. South America, with its rich flora, harbors a variety of herbs used in healing remedies and more recently in phytomedicine. *Baccharis coridifolia* [5] (also known in Uruguay as “mío-mío”) has been long known as a poisonous plant affecting livestock in Uruguay [6]. Herein, we first report on antileukemic activity of OST-01, a novel NP extracted from *Baccharis coridifolia*, on AML “bulk” and LSC-enriched blasts. While the OST-01 mechanism of action (MOA) is likely multifaceted given the plethora of diverse molecular species generally contained in NPs, we observed that disruption of c-Myc-dependent ribosome biogenesis appears to be central to its antileukemic activity.

**Fig. 1:**
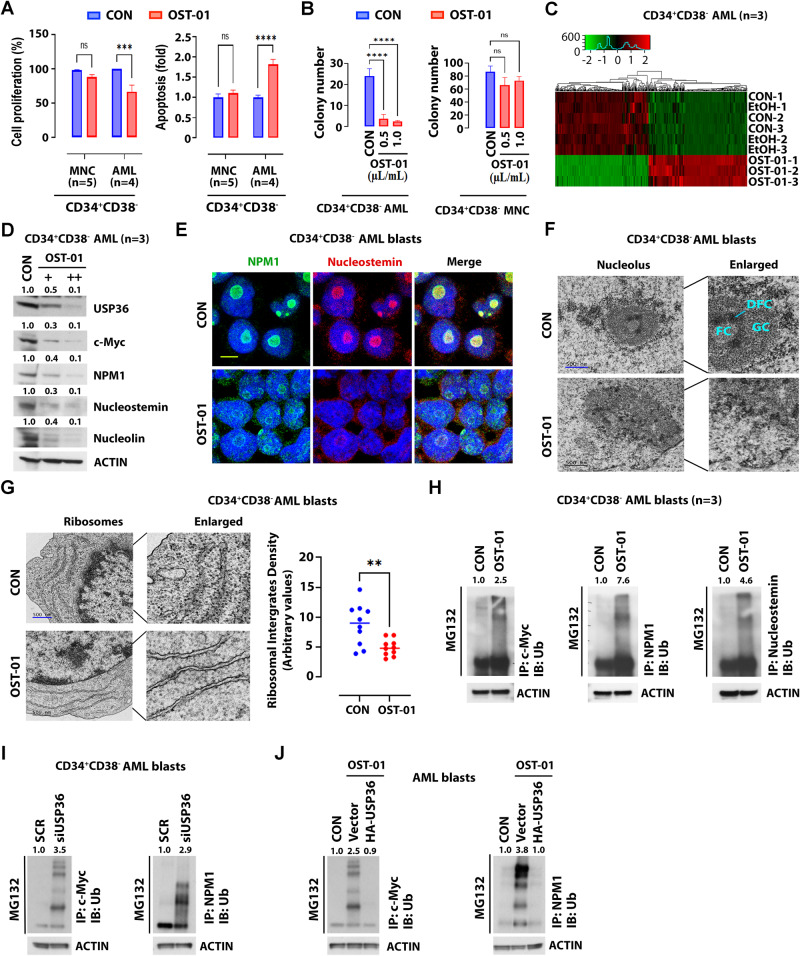
Effects of OST-01 on proliferation and apoptosis of AML cell lines and LSC-enriched AML blasts. **A** Effects of OST-01 on proliferation and apoptosis of LSC-enriched AML blasts. CD34 + CD38- cells were isolated from primary MNCs (*n* = 5) or AML blasts (*n* = 4). Left, levels of cell proliferation. Right, levels of apoptosis. **B** Effects of OST-01 on colony forming of LSC-enriched AML blasts. CD34 + CD38- AML blasts or MNCs (2 × 10^5^ cells/ml, *n* = 3) were treated with 1 µL/mL of ethanol control or indicated dose of OST-01 for 24 h before plating on methylcellulose. After 14 days, colonies were images under light microscope and counted, *N* =  2, data are presented as mean ± SE, with triplicate determination. Number of colonies are presented as bar graph. Asterisk indicates statistically significant difference based on unpaired *t* test analysis. **C** Heatmap showing change of gene expression in primary CD34 + CD38- AML blasts treated with ethanol control or OST-01 (1 µL/mL) for 24 h (each group, *n* = 3). **D** Effects of OST-01 on expression of deubiquitinase USP36 and nucleoprotein. Primary CD34 + CD38- AML blasts were treated with ethanol control or dose dependent of OST01 (+0.5 µL/mL; ++1 µL/mL). Immunoblotting of indicated antibodies are shown. Quantification of protein expressions are shown on top. **E** Effects of OST-01 on cellular distribution of nucleolar proteins. Primary CD34 + CD38- AML blasts were treated with ethanol control or OST-01 (1 µL/mL) for 24 h, following by staining with anti-NPM1 and anti-nucleostemin antibodies. The images were taken under a confocal microscope. Scale bar, 10 µm. **F** Effects of OST-01 on nucleolus structure. Primary CD34 + CD38- AML blasts were treated with ethanol control or OST-01 (1 µL/mL) for 24 h. Transmission electron microscope (TEM) was performed to image nucleolus structure. Enlarged images are shown on the right. FC fibrillary center; DFC dense fibrillar component; GC granular component. **G** Effects of OST-01 on number of ribosome upon treatment of primary CD34 + CD38- AML blasts with ethanol control or OST-01 (1 µL/mL) for 24 h. Left, TEM images of ribosome. Right, quantification of ribosome levels. Asterisk indicates significantly different based on unpaired t test analysis. **H** Effects of OST-01 on ubiquitination of nucleoprotein. Primary CD34 + CD38- AML blasts were treated with ethanol control or OST-01 (1 µL/mL) for 24 h. The cell lysate was immunoprecipitated with anti-c-Myc, anti-NPM1 or anti-nucleostemin and immunoblotted with anti-Ubiquitin (Ub) antibodies. **I** Effects of USP36 knockdown on ubiquitination of c-Myc and NPM1 protein. Primary CD34 + CD38- AML blasts were transfected with scramble siRNA control or USP36 siRNA (40 nM) for 24 h. The cell lysate was immunoprecipitated with anti-c-Myc and anti-NPM1 and immunoblotted with anti-Ubiquitin (Ub) antibodies. **J** Effects of USP36 overexpression on OST-01 regulated c-Myc and NPM1 ubiquitination. Primary CD34 + CD38- AML blasts were transfected with vector control or HA-Flag-USP36 for 12 h, following by treatment with OST-01 (1 µL/mL) combined with MG132 (10 µM) for 24 h. The cell lysate was immunoprecipitated with anti-c-Myc and anti-NPM1 and immunoblotted with anti-Ubiquitin (Ub) antibodies.

OST-01 treatment on dose dependent for 24 h significantly inhibits proliferation and colony formation and induces apoptosis in representative AML cell lines (MV-4-11, KG-1a, Kasumi-1 and HL-60), primary CD34 + CD38- blasts (enriched for LSCs; see Table S1 for molecular features), but not normal CD34 + CD38- mononuclear cells (MNCs) [enriched for hematopoietic stem cells (HSC)] (Fig. 1A, B, Fig. S1), suggesting that OST-01 was cytotoxic to AML blasts, but spared normal HSCs. To gain mechanistic insights into the antileukemic activity of OST-01, we performed RNA-seq in primary CD34 + CD38- AML cells (*n* =  3) treated with OST-01 or vehicle (both at 1 µL/mL) for 24 h. Exposure to OST-01 resulted in 1330 differentially expressed genes (790 upregulated and 540 downregulated) (Fig. 1C). The oncogene MYC was among the top five downregulated genes and the gene set enrichment analysis (GSEA) indicated downregulation of gene sets enriched for c-Myc targets, ribonucleoprotein complex, and ribosome biogenesis in OST-01 treated cells (Fig. S2A–D**)**. Given that c-Myc regulates nucleolar assembly and aberrant expression of c-Myc promotes cancer cell growth through increase of ribosomal biogenesis [7–9], we hypothesized that OST-01-disrupted c-Myc regulated nucleolar structure and ribosome biogenesis is one of its antileukemic MOAs. A twenty-four-hour exposure to OST-01 resulted in a significantly decreased levels of c-Myc and other nucleolar proteins i.e., NPM1, nucleostemin and nucleolin, (Fig. 1D, E and Fig. S2E) and disruption of the normal nucleolar architecture in HL-60 cells and CD34 + CD38- AML blasts (Fig. 1F and Fig. S2F). Accordingly, we observed a significantly decreased number of ribosomes in OST-01-treated cells (Fig. 1G and Fig. S2G). Interestingly, OST-01 significantly inhibited USP36, a nucleolar protein deubiquitinase [10, 11], which lead to ubiquitination and degradation of c-Myc, NPM1, and nucleostemin (Fig. 1D and H). Accordingly, USP36 knock-down increased c-Myc and NPM1 ubiquitination (Fig. 1I). In contrast, USP36 overexpression rescued OST-01-induced c-Myc and NPM1 ubiquitination (Fig. 1J). Thus, taken altogether, these results indicate disruption of de-ubiquitination of c-Myc and other nucleolar proteins as a possible OST-01 antileukemic MOA.

Notably, we have initiated the OST-01 purification workflow to extract, purify, and identify the principal molecules responsible for OST-01’s pharmacologic activity (Fig. S3A, details in methods). Beginning with flash chromatography, we obtained 60 OST-01 fractions (F1-60) and tested their bioactivity on MV-4-11 cells. The most active fraction inducing MV-4-11 apoptosis underwent reversed-phase semi-preparative HPLC for further purification. The purity of active compounds was assessed in sub-fractions lasting between 18 to 22 min (Fig. S3B, C). An LC-MS/MS analysis was performed (Fig. S3D), and the raw data were searched against plant NP databases, including 14 publicly available databases on plant natural products (details in methods). One of the hits that emerged from our analysis was (2E)-21-Hydroxy-2-henicosenoic acid (2E-21). This compound exhibited antileukemic activity and recapitulated the OST-01 pharmacologic effects, including the disruption of nucleolar structure, reduced of number ribosomes, and decreased levels of USP36, c-Myc, and NPM1 (Fig. S3E–I).

**Fig. 2:**
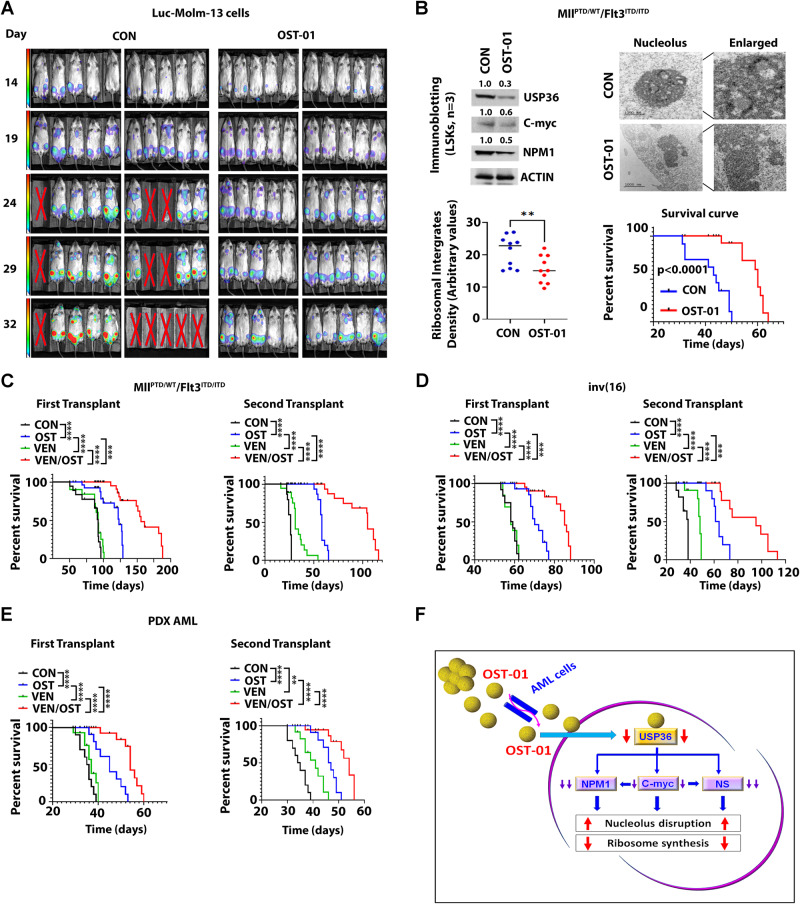
Antileukemic activities of OST-01 in vivo. **A** Effects of OST-01 on leukemic cell growth in vivo and AML mouse survival. Luc-Molm-13 cells (1.0 × 10^6^) were intravenously injected in NSG mice. After 7 days of injection, mice were treated with ethanol control or OST-01 [1 µL/g/BID, oral gavage, continuously till the euthanized endpoint]. Leukemia growth was determined 14 days after the start of treatment using bioluminescence imaging. N =  10 mice per group. The survival curve of treated mice is shown on Fig. S4B. **B** Effects of OST-01 on leukemic cell growth in vivo using Mll^PTD/WT^/Flt3^ITD/ITD^ AML mouse model. Mll^PTD/WT^/Flt3^ITD/ITD^ AML cells (1 × 10^6^ cells/mouse) were transplanted into WT mice to generate a cohort of AML-bearing mice, which were randomly divided into two groups and treated with ethanol control [1 µL/g/BID, oral gavage, (*n* =  8)] or OST-01 [1 µL/g/BID, oral gavage, (*n* =  10)] for 21 days. Leukemia burden as determined by number of LSKs and spleen size was shown on Fig. S4D. LSKs were isolated from the treated mice. Top, immunoblotting of USP36, c-Myc and NPM1 protein (left) and TEM images of nucleolus (right). Bottom, ribosome levels in LSKs (left) and Kaplan–Meier survival curve of primary transplanted leukemic mice (right). Ethanol control [blue line; *n* =  10; median survival (MS) 44 days] or OST-01 (red line; *n* =  10; MS 60 days). **C** and **D** Synergistic effects of OST-01 and VEN in vivo. **C** Mll^PTD/WT^/Flt3^ITD/ITD^ AML cells (0.5 × 10^6^ cells/mouse) were transplanted into WT mice to generate a cohort of AML bearing mice, which were randomly divided into 4 groups (*n* =  15) and treated with ethanol control (1 µL/g/BID, oral gavage, continuously till the euthanized endpoint), OST-01 (1 µL/g/BID, oral gavage, continuously till the euthanized endpoint), VEN (50 mg/kg, oral gavage, 21 days), or combination of OST-01 and VEN at the same doses of single agents. (Left) Kaplan–Meier survival curve of primary transplanted leukemic mice treated with ethanol control (black line; MS 92 days), OST-01 (blue line; MS 123 days), VEN (green line; MS 92 days), or OST/VEN (red line; MS 156 days). (Right) Kaplan–Meier survival curve of secondary transplanted leukemic mice treated with ethanol control (black line; MS 26 days), OST-01 (blue line; MS 58 days), VEN (green line; MS 31 days), or OST/VEN (red line; MS 105 days). **D** Inv(16) AML mice. (Left) Kaplan–Meier survival curve of primary transplanted leukemic mice treated with ethanol control (black line; MS 58.5 days), OST-01 (blue line; MS 70 days), VEN (green line; MS 58 days), or OST/VEN (red line; MS 85 days). (Right) Kaplan–Meier survival curve of secondary transplanted leukemic mice treated with ethanol control (black line; MS 38 days), OST-01 (blueline; MS 62.5 days), VEN (green line; MS 48 days), or OST/VEN (red line; MS 95 days). **E** FLT3-WT PDX AML mice. (Left) Kaplan–Meier survival curve of primary PDX model treated with ethanol control (black line; MS 35.5 days), OST-01 (blue line; MS 45 days), VEN (green line; MS 37 days), or OST/VEN (red line; MS 54 days). (Right) Kaplan–Meier survival curve of secondary transplanted leukemic mice treated with ethanol control (black line; MS 34.5 days), OST-01 (blue line; MS 47 days), VEN (green line; MS 41 days), or OST/VEN (red line; MS 54 days). **F** Schematic model mechanism of action of OST-01 in AML cells. OST-01 inhibits deubiquitinase USP36 expression, leading to downregulation of nucleolar proteins including c-Myc, nucleophosmin (NPM1), and nucleostemin (NS). This consequently disrupts nucleolus structure and inhibits ribosome synthesis.

To test OST-01 in vivo, we injected 0.5 × 10^6^ luciferase-expressing FLT3-ITD+ Molm-13 AML cells (Luc-Molm13 cells) into immunodeficient NSG mice and treated them with either OST-01 or vehicle (ethanol) [1 µL/g, oral gavage, BID] until they achieved a euthanized endpoint (Fig. S4A). Tumor growth was monitored by bioluminescence imaging. OST-01-treated mice demonstrated a significant reduction of the leukemia burden (Fig. 2A) and increase of survival compared with vehicle-treated mice (Fig. S4B; *p* < 0.0001).

We confirmed these results in another aggressive murine AML model, the Mll^PTD/WT^/Flt3^ITD/ITD^ B6 mouse (Fig. S4C) [12]. The OST-01-treated mice had a significantly reduced disease compared with vehicle-treated mice as shown by significantly smaller spleens and decrease of BM Lin-cKit+Sca+ (LSK; identified as stem and progenitor population; Fig. S4D). We also observed decreased levels of USP36 and nucleolar protein including c-Myc and NPM1, nucleolar disruption, and reduced number of ribosomes in BM LSKs isolated from OST-01-treated mice compared with those isolated from vehicle-treated mice (Fig. 2B and Fig. S4E). Importantly, OST-01-treated mice also survived significantly longer than the vehicle-treated controls (*p* < 0.0001; Fig. 2B, lower right).

To investigate the safety of OST-01 in vivo, we administered escalating dosage of OST-01 to normal black B6 mice. Male and female mice were treated with 0.5 uL/g, 1.0 uL/g, or 1.5 uL/g of OST-01 or vehicle, BID, by oral gavage for 7, 28, or 56 days (each group, *n* = 5; Fig. S5A). Compared to vehicle-treated mice, we observed no relevant changes in weight (Fig. S5B), behavior, or complete blood count (CBCs) in OST-treated mice (Fig. S6A). H&E-stained sections of brain, heart, kidney, liver, lung, and spleen harvested at each time point also showed no difference in OST-01-treated vs vehicle-treated mice including those which received highest dose 1.5 μL/g, BID, for 56 days (Fig. S6B, C).

We next tested the activity of OST-01 in combination with venetoclax (VEN), a selective BCL-2 inhibitor broadly used in combination with hypomethylating agents or other chemotherapeutics in AML patients [13], in CD34 + CD38- AML blasts. Compared with VEN or OST-01 alone, OST-01/VEN had synergistic antileukemic effects, as demonstrated by inhibition of proliferation and colony forming and increasing apoptosis (Fig. S7A–C). For in vivo studies, normal B6 WT mice transplanted with 0.5 × 10^6^ Mll^PTD/WT^/Flt3^ITD/ITD^ BM MNCs and treated with either vehicle, OST-01, VEN, or OST-01/VEN (Fig. S7D). While OST-01-treated mice lived significantly longer than VEN- and vehicle-treated mice, OST-01/VEN-treated mice lived significantly longer than those treated with single agents or vehicles (Fig. 2C, left). In secondary transplant experiments, the recipients of BM MNCs from mice treated with OST-01/VEN also lived significantly longer than the recipients of BM MNCs from mice treated with either agent alone or vehicle, suggesting activity of OST-01 on LSCs (Fig. 2C, right).

We corroborated these results in a third AML murine model, the Cbfb-MYH11 knock-in mouse that recapitulates the human inv(16) AML (Fig. S7D) [14]. OST-01-treated mice lived significantly longer than VEN- or vehicle-treated mice while OST-01/VEN-treated mice lived longer than those treated with either agent alone or vehicle (Fig. 2D, left). In secondary transplant experiments, the recipients of BM MNCs from mice treated with OST-01/VEN lived significantly longer than the recipients from of BM MNCs from mice treated with either agent alone or control (Fig. 2D, right).

We further evaluated the in vivo efficacy of OST-01/VEN combination in a FLT3-WT AML patient-derived xenograft (PDX) model. The PDX mice were treated as described above. At the end of treatment, we observed OST-01/VEN-treated mice have smaller spleens (Fig. S7E) and lower AML burden compared to mice treated with single agents or control (Fig. S7F, left). In secondary transplant experiments, recipients of BM MNCs from OST-01/VEN treated mice had reduced AML burden compared to recipients of BM MNCs from mice treated with either agent alone or vehicle (Fig. S7F, right). Furthermore, prolonged survival was seen in the recipients of BM MNCs from PDX mice treated with OST-01/VEN compared to recipients of BM MNCs from mice treated with either agent alone or vehicle (Fig. 2E), suggesting activity of OST-01 also on human LSCs.

In summary, we first report here a significant antileukemic activity of a novel NP, OST-01, alone or in combination with VEN in murine and human AML models through inhibition of c-Myc-dependent ribosome biogenesis (Fig. 2F). While our results strongly suggest that the decreased of ribogenesis is mediated directly by the activity of one or more OST-01 molecules on c-Myc and the ribosome machinery, the possibility that this may instead be an indirect effect related to a general OST-01-mediated cell proliferation inhibition cannot be entirely excluded. Nevertheless, in support of a more direct OST-01 effect was the observation that OST-01 was toxic to AML cells but spared normal HSCs. While the molecular basis for this preferential activity of OST-01 on leukemic cells over normal HSCs remains to be fully elucidated, “addiction” of AML cells to c-Myc upregulation and in turn to high level of ribosome biogenesis may explain this finding. Full molecular, structural, and functional characterizations of OST-01 to identify more active principle(s) and other IND-enabling GLP-toxicology studies are underway for a rapid transition of OST-01 from the bench to the clinic.

### Supplementary information

Supplemental Information

Figure S1

Figure S2

Figure S3

Figure S4

Figure S5

Figure S6

Figure S7

## Data Availability

All datasets generated during this study are available from the corresponding author on reasonale request.

## References

[1] Dick JE (2005). Acute myeloid leukemia stem cells. Ann N Y Acad Sci.

[2] Atanasov AG, Waltenberger B, Pferschy-Wenzig EM, Linder T, Wawrosch C, Uhrin P (2015). Discovery and resupply of pharmacologically active plant-derived natural products: a review. Biotechnol Adv.

[3] Mann J (2002). Natural products in cancer chemotherapy: past, present and future. Nat Rev Cancer.

[4] Harvey AL, Edrada-Ebel R, Quinn RJ (2015). The re-emergence of natural products for drug discovery in the genomics era. Nat Rev Drug Discov.

[5] Abad MJ, Bedoya LM, Bermejo P. Chapter 14 - Essential oils from the Asteraceae family active against multidrug-resistant bacteria. In: Rai MK, Kon KV, editors. Fighting multidrug resistance with herbal extracts, essential oils and their components. San Diego: Academic Press; 2013. p. 205–21.

[6] Freitas PR, de Araújo ACJ, dos Santos Barbosa CR, Muniz DF, Rocha JE, de Araújo Neto JB (2020). Characterization and antibacterial activity of the essential oil obtained from the leaves of Baccharis coridifolia DC against multiresistant strains. Microb Pathog.

[7] Destefanis F, Manara V, Bellosta P (2020). Myc as a regulator of ribosome biogenesis and cell competition: a link to cancer. Int J Mol Sci.

[8] van Riggelen J, Yetil A, Felsher DW (2010). MYC as a regulator of ribosome biogenesis and protein synthesis. Nat Rev Cancer.

[9] Brown IN, Lafita-Navarro MC, Conacci-Sorrell M (2022). Regulation of nucleolar activity by MYC. Cells.

[10] Sun XX, Sears RC, Dai MS (2015). Deubiquitinating c-Myc: USP36 steps up in the nucleolus. Cell Cycle.

[11] Endo A, Matsumoto M, Inada T, Yamamoto A, Nakayama KI, Kitamura N (2009). Nucleolar structure and function are regulated by the deubiquitylating enzyme USP36. J Cell Sci.

[12] Zhang B, Nguyen LXT, Li L, Zhao D, Kumar B, Wu H (2018). Bone marrow niche trafficking of miR-126 controls the self-renewal of leukemia stem cells in chronic myelogenous leukemia. Nat Med.

[13] DiNardo CD, Jonas BA, Pullarkat V, Thirman MJ, Garcia JS, Wei AH (2020). Azacitidine and venetoclax in previously untreated acute myeloid leukemia. N Engl J Med.

[14] Zhang L, Nguyen LXT, Chen YC, Wu D, Cook GJ, Hoang DH (2021). Targeting miR-126 in inv(16) acute myeloid leukemia inhibits leukemia development and leukemia stem cell maintenance. Nat Commun.

