# Supplementary material for: OST-01, a natural product from Baccharis coridifolia, targets c-Myc-dependent ribogenesis in acute myeloid leukemia
Source: Leukemia. 2024 Jan 17;38(3):657–62. doi: 10.1038/s41375-024-02146-5 (PMC10912030; doi:10.1038/s41375-024-02146-5)
Supplement: Supplementary file 1 — Supplemental Information [file 41375_2024_2146_MOESM1_ESM.docx]

**SUPPLEMENTAL INFORMATION**

1. **Supplementary Materials and Methods** (Page 2 to Page 8)
2. **Supplementary References:** (Page 8)
3. **Supplementary Tables:** (Page 9 to Page 10)

**+ Table S1.** Characteristics of patient samples

**+ Table S2.** The target sequences for siRNAs

**+ Table S3.** List of antibodies used for IP, IB, and IF analysis

1. **Supplementary Figures Legends** (Page 11 to Page 16)

+ **Figure S1.** Effects of OST-01 on proliferation and apoptosis of AML cell lines, HSC-enriched normal MNCs and LSC-enriched AML blasts

+ **Figure S2.** Effects of OST-01 on USP36-regulated nucleolus structure and ribosome synthesis

+ **Figure S3.** Identification and characterization of principal molecule in OST-01

+ **Figure S4.** Antileukemic activities of OST-01 *in vivo*

**+ Figure S5.** Safety of OST-01 *in vivo-*weight and behavior

**+ Figure S6.** Safety of OST-01 *in vivo-*blood count and pathology analysis

**+ Figure S7.** Synergistic effects of OST-01 and venetoclax *in vitro* and *in vivo*

1. **Supplementary Figures**

**Supplementary Materials and Methods**

**OST-01 manufacture**

OST-01 is a natural product produced by Ostentus Therapeutic Incorporation. To prepare OST-01, leaves and stems from *Baccharis coridifolia* were harvested and washed in distilled water, dried with paper towels, and cut into small pieces. The plant material was then macerated with 95% ethanol in the dark at 15 - 20°C for 30 days. The pressed extract was then filtered, and the eluent was considered an extract comprising one or more anti-oncogenic phytochemicals and was designated as OST-01 (patent #UEOST1-0001US).

**Human samples**

Normal HSCs and AML samples were obtained from healthy donors and patients at the City of Hope National Medical Center (COHNMC), under the City of Hope Institutional Review Board approved banking protocols (#06229, #03162, #07047 or #18067) in accordance with assurances filed with and approved by the Department of Health and Human Services and meeting all requirements of the Declaration of Helsinki. Written informed consent was obtained from donors (#06229) or patients (#03162, #07047 or #18067) prior to specimen acquisition. Patient characteristics of primary AML samples are listed in Supplementary Table S1.

**Isolation of mononuclear cells from patient samples**

Each patient specimen was transferred to a 50 mL conical tube and the volume was brought up to 25 mL using warm 1x Dulbecco’s phosphate buffered saline (DPBS) with 2% FBS. The specimen was layered on the top of 20 mL Ficoll-Paque Plus in a 50 mL conical tube. Then, the tube was centrifuged at 300 g for 32 minutes without break. The layer containing PBMC, and plasma was carefully transferred to a 50 mL conical tube and the volume was brought up to 50 mL with warm 1x DPBS. The tube was then centrifuged at 2400 rpm for 8 minutes. The supernatant was discarded, and the pellet was resuspended in 10 mL of warm DPBS (1x). Cell number and viability were determined, and the sample was frozen.

**Mice**

All transplant was performed via tail vein intravenous (i.v.) injection with Molm-13-luciferace model and human primary FLT3 wild-type AML blasts into sublethal irradiated (XRAD 320-Precision X-Ray; 2.0 Gy) 6-8-week-old syngenic NSG mice, Mll^PTD/WT^/Flt3^ITD/ITD^ and Cbfb-MYH11 knock-in AML into sublethal irradiated (XRAD 320-Precision X-Ray; 4.5 Gy) 6-8-week-old syngenic C57BL/6 mice. *In vivo* administration of OST-01 or ethanol control was performed by oral gavage injection (1 µL/g, BID). All mice were maintained in an Association for Assessment and Accreditation of Laboratory Animal Care accredited animal facility and all experimental procedures were performed in accordance with federal and state government guidelines and established institutional guidelines and protocols approved by the Institutional Animal Care and Use Committee at City of Hope.

**Cell cultures, Plasmid and Chemicals**

MV-4-11, KG-1a, Kasumi-1 and HL-60 cells were purchased from the American Type Culture Collection and maintained in IMDM (Iscove’s Modified Dulbecco’s Medium) or RPMI (Roswell Park Memorial Institute) medium supplemented with 10% FBS and 100 units of penicillin/streptomycin at 37°C with 5% CO_2_ and high humidity. Human cell lines purchased from ATCC more than 6 months prior to submission of this manuscript and not frozen at an early passage were authenticated using ATCCs’ human short tandem repeat (STR) DNA profiling authentication service. Morphology of cell lines was monitored routinely, and cell lines were routinely subjected to mycoplasma detection using a mycoplasma detection kit (Roche, Germany). Flag-HA-USP36 was a gift from Wade Harper (Addgene plasmid #22579) (1). Venetoclax and MG132 were from Selleckchem (Houston, TX). (2E)-21-Hydroxy-2-henicosenoic acid was synthesized at City of Hope.

**Cell proliferation assay**

A water-soluble tetrazolium salt (WST-1; Cat #5015944001, Millipore Sigma) assay was performed to quantify the antileukemic effects of OST-01 on AML cell growth. Cells were plated at density of 100,000 cells per well of 96-well plate and treated with different dose of OST-01 for 24 hours in 37°C incubator. Then, 10 μL of WST-1 solution was added to 100 μL of culture media and the plate was incubated at 37°C incubator for 2 to 3 hours. The metabolic product of WST-1, Formazan, was measured using a multi-well spectrophotometer (wavelength, 450 nm).

**Colony forming assay**

LSC potential was evaluated by colony forming assay. Briefly, 5×10^3^ OST-01 and vehicle-treated cells were added to 1.5 mL H4436 Methocult (Stem Cell Technologies) and mixed by vortex. Then, the cells were transferred to 6-well plate for culture at 37°C, and colonies were assessed at day 12 - 14 days of culture.

**Transmission Electron Microscope (TEM)**

OST-01 or vehicle-treated cells were fixed with 2.5% glutaraldehyte, 0.1M Cacodylate buffer (Na(CH_3_)_2_AsO_2_ ·3H_2_O), pH7.2, at 4°C. Standard sample preparation for TEM was followed including post-fixation with osmium tetroxide, serial dehydration with ethanol, and embedment in Eponate. Ultra-thin sections (70 nm thick) were acquired by ultramicrotomy, post-stained, and examined on an FEI Tecnai 12 transmission electron microscope equipped with a Gatan OneView CMOS camera. TEM images were taken at nominal 11,000 x magnification.

**Ubiquitin assay**

OST-01 or vehicle-treated cells were collected and subsequently lysed in RIPA buffer containing 10 mM protease inhibitor cocktail (Thermo Scientific). Then, 500 µg of cell lysate were incubated with indicated antibodies for overnight at 4°C. 50 µl of Protein A/G agarose beads (Santa Cruz) were added and the mixture was inverted for 3 hours at 4°C. Immunoprecipitated complex was separated on NuPAGE 4-12% gradient gels (Invitrogen) and immunoblotted with anti-Ubiquitin antibody.

**DNA fragmentation assay**

OST-01 or vehicle-treated cells were lysed on ice for 60 min in 500 μL lysis buffer containing 0.02% SDS, 1% Nonidet P-40 and 0.2 mg/mL proteinase K in PBS. Genomic DNA was extracted using the phenol/chloroform method. The pellet was dissolved in 50 μL of TE buffer (supplemented with 10 mg/mL RNase) for 2 h at 37 °C. A total of 10 μg of DNA was loaded on a 2% agarose gel and visualized under UV light.

**Synthetic small interfering RNA (siRNA) oligonucleotides**

### The siGENOME SMARTpool for siRNA of USP36 were purchased from Horizon Discovery. Scrambled control RNA (siSCR) was used as a control. The target sequences for siRNAs are shown in Supplementary Table S2.

**RNA-seq and Gene set enrichment analysis (GSEA)**

For high resolution genomic profiling (mRNA-seq) to evaluate mRNA profiles, sequencing was performed on an Illumina Hiseq 2500. Reads were trimmed to remove poly(A) tail and Illumina adapter using Trimmomatics, then aligned to Human Genome Assembly GRCh38.p14 using Bowtie2 v2.5.1 with default settings. The expression level of ensemble genes was counted using RSEM v1.3.3. Custom R scripts and Bioconductor packages “edgeR” were used for data normalization and inter-group comparisons. GSEA v4.3.2 were used to identify Gene Ontology (GO) and canonical pathways (downloaded from Msigdb v7.0) that are altered upon OST-01 treatment vs. ethanol control.

**Immunoblotting analysis**

Cells were washed and harvested in ice-cold PBS and subsequently lysed in RIPA buffer containing 10 mM protease inhibitor cocktail (Thermo Scientific). For immunoblotting, 50 µg of each cell lysate was separated on NuPAGE 4-12% gradient gels (Invitrogen) and immunocomplexes were visualized with enhanced chemiluminescence reagent (Thermo Scientific). The list of antibodies is presented in Supplementary Table S3.

**Immunocytochemistry**

Cells were collected, washed in ice-cold PBS and mounted on glass slides using a Cytocentrifuge (CytoSpin4, 600 rpm, 10 minutes). Cells were then washed with PBS, fixed in 4% paraformaldehyde for 15 minutes and permeabilized in 0.5% Triton X-100 for 15 minutes. Non-specific epitopes were blocked with 5% bovine serum albumin (BSA) for 30 minutes. Primary antibodies are listed in Supplementary Table S3. Secondary anti-mouse/rabbit/goat-Alexa 594/488/647 goat antibodies were purchased from Thermo Scientific. Cell images were acquired using a Zeiss confocal laser-scanning-microscope (Zeiss LSM 800). Nuclei were counterstained with ProLong Gold Antiface with DAPI (Molecular Probes, Invitrogen).

**Annexin-V staining**

The Annexin-V and DAPI double staining method was used to evaluate apoptosis by flow cytometry. Cells were harvested and washed twice with Annexin-V binding buffer (BD Bioscience) and resuspended in 100 μL of the same buffer containing Annexin-V APC (BD Bioscience). Cells were then incubated in the dark at room temperature for 15 min, washed again and resuspended in 300 μL of buffer. DAPI (Sigma-Aldrich) was added immediately prior to analysis with a LSR II flow cytometer (BD Bioscience).

**Cell isolation and flow cytometry**

Using a mortar and pestle, BM mononuclear cells were collected from femurs, tibias and pelvis. For fluorescence activated cell-sorting (FACS) analyses, cells were stained with fluorescently labeled antibodies in PBS with 0.5% BSA for 15 min at 4ºC. Flow cytometry was performed using a 5-laser, BD LSRFortessa™ X-20 cell analyzer. Antibodies used included hCD33 and hCD45 purchased from BioLegend. For isolating mouse leukemic stem cells/hematopoietic stem and progenitor cells (mHSPCs), lineage-negative (Lin-) cells were firstly enriched using EasySep™ negative selection reagents (STEMCELL Technologies, 18000) and then LSK (Lin−cKit+Sca1+) cells were subsequently sorted on a 5-Laser, BD FACSAria Fusion Cell Sorter. For isolating human primary blast/hematopoietic stem and progenitor cells (hHSPCs), which are immunophenotypically CD34+CD38-, cells (cell sources: Ficol-separated cells from BM or CB) were isolated using Human CD34+CD38- Cell Isolation Kit (Cat# 130-114-822, Miltenyi Biotec). Briefly, CD34+ cells were first stained with CD34-microbeads and isolated by magnetic separation and then further enriched by depleting CD38+ cells. Acquired data was analyzed by Flowjo software 10.6.1.

**Procedure for isolating principal molecules of OST-01**

OST-01 were fractionated using silica gel flash chromatography (CombiFlash NextGen 300) and fractions were tested for bioactivity using annexin V staining. Active fractions were then further subjected to reversed phase semi-preparative HPLC separation (Agilent 1100 HPLC) coupled to a photodiode array detector. Purity is assessed by HPLC and characterized by their molecular weight, molecular formula as well as chemical structure. High resolution mass spectrometry (Thermo Orbitrap Lumos Tribrid) was employed to elucidate molecular weight (MW) and elemental composition (EC) of candidate molecules. To identify candidate molecules, LC-MS/MS analysis was performed on partially purified compounds with bioactivity. The raw data was searched against plant natural product database including Carotenoids Database, ChEBI, ChEMBL, ChemSpiderman, Crystallography Open Database (COD), Dr. Parvinder Pal Singh-Medicinal Chemistry Division- CSIR-Indian Institute of Integrative Medicine, DrugBank, DTP-eCrystals, Food and Agriculture Organization of the United Nations, PubMed-Arita Lab 6549 Flavonoid Structure Database, GC Orbitrap Flavor and Fragrances Compound Database, natural Products Atlas 2020, and mzCloud using Compound Discoverer 3.2 (Thermo).

**Statistical analysis**. To compare the means of 2 groups, results were generally compared by using unpaired, two-tailed student’s t-test, with values from at least 2 independent experiments with triplicate determination, unless otherwise stated. Data are presented as mean ± standard error (S.E.), as indicated. P<0.05 was considered statistically significant; ns = not significant. *, p≤0.05, **, p≤0.01, ***, p≤0.001, ****, p≤0.0001. All statistical analyses were conducted using SigmaPlot 12.5 (Systat Software, Chicago, Illinois). All statistical tests were two-sided.

**Supplementary References**

1. Sowa ME, Bennett EJ, Gygi SP, Harper JW. Defining the human deubiquitinating enzyme interaction landscape. Cell. 2009;138(2):389-403.

**Supplementary Table S1. Characteristics of patient samples**

| **Sample ID** | **Sample Type** | **Disease Status** | **Cytogenetic** | **Mutations** | **WBC (10^3^/μl)** | **PB Blasts (%)** | **BM Blasts (%)** |
| --- | --- | --- | --- | --- | --- | --- | --- |
| AML01 | PB | New Diagnosis | Normal | FLT3-ITD Pos, CEBPA Pos | 76.8 | 84 | 75 |
| AML02 | BM | Refractory | Complex | TET2 Pos | 105 | 34 | 15 |
| AML03 | PB | New Diagnosis | Normal | FLT3-ITD Pos | 22 | 83 | 90 |
| AML04 | BM | New Diagnosis | Normal | NPM1 Pos | 33 | 87 | 90 |
| AML05 | BM | Relapsed | Complex | NPM1 Pos, FLT3-ITD Pos | 46.4 | 90 | >90 |
| AML06 | BM | Relapsed | Normal | FLT3-ITD Pos | 15 | 6 | 19 |
| AML07 | BM | New Diagnosis | Normal | CEBPA Pos | 33 | 70 | 60 |
| AML08 | PB | Relapsed | Complex | FLT3-ITD Pos, NPM1 Pos | 46.4 | 66 | >90 |
| AML09 | PB | Relapsed | Normal | FLT3-ITD Neg, NPM1 Neg, IDH1 Neg | 29 | 91 | >90 |
| AML10 | PB | New Diagnosis | Normal | IDH2 Pos | 1.4 | 5 | >90 |
| AML11 | PB | New Diagnosis | Normal | FLT3-ITD Pos, NPM1 Pos | 33 | 87 | 90 |
| AML12 | BM | Relapsed | Complex | NPM1 Pos | 20.7 | 59 | 90 |
| AML13 | PB | New Diagnosis | Normal | FLT3-ITD Pos, NPM1 Pos | 7.6 | 20 | 62 |
| AML14 | BM | New Diagnosis | Normal | DNMT3A, IDH2 Pos, NPM1 Pos | 151.7 | 91 | >95 |
| AML15 | PB | New Diagnosis | Complex | FLT3-ITD Pos, NPM1 Pos | 33 | 80 | 60 |

**Supplementary Table S2. The target sequences for siRNAs**

| No | Name | Target Sequence |
| --- | --- | --- |
| 1 | siUSP36 | 5’-CCUUAAAGAGCAAAUAUGU-3’  5’-UUCUCAUGUUUCAUAGACA-3’  5’-CCUUGGUCCAUCAAAUUUU-3’  5’-CUGUCAUGUAUGGACUCUA-3’ |

**Supplementary Table S3. List of antibodies used for IP, IB, and IF analysis**

| No | Antibody name | Information |
| --- | --- | --- |
| 1 | Anti-PARP antibody | Cat# 9542, Cell Signaling |
| 2 | Anti-p53 antibody | Cat# 9282, Cell Signaling |
| 3 | Anti-pH2AX antibody | Cat# 9718, Cell Signaling |
| 4 | Anti-Caspase 3 cleavage antibody | Cat# 9661, Cell Signaling |
| 5 | Anti-ACTIN antibody | Cat# sc-47778, Santa Cruz |
| 6 | Anti-USP36 antibody | Cat# NBP2-55536, Novus |
| 7 | Anti-C-myc antibody | Cat# sc-40, Santa Cruz |
| 8 | Anti-NPM1 antibody | Cat# 3542, Cell Signaling |
| 9 | Anti-nucleostemin antibody | Cat# sc-166460, Santa Cruz |
| 10 | Anti-Ub antibody | Cat# 07-357, Millipore |

**Supplementary Figure Legends**

**Figure S1. Effects of OST-01 on proliferation and apoptosis of AML cell lines, HSC-enriched normal MNCs and LSC-enriched AML blasts. A** Effect of OST-01 on cell viability of leukemic cell lines. Cell lines MV4-11, KG-1a, Kasumi-1, and HL-60 (10^5^ cells) were incubated with indicated doses of OST-01 for 24 hours. Cell viability was determined by WST-1 proliferation assay. IC50s value: MV-4-11, 3.47 µL/mL; KG-1A, 0.99 µL/mL; Kasumi-1, 1.23 µL/mL; HL-60, 1.39 µL/mL. **B-D** Effect of OST-01 on proliferation and apoptosis of leukemic cell lines. Cell lines MV4-11, KG-1a, Kasumi-1, and HL-60 (10^5^ cells) were incubated with 1 µL/mL OST-01 for 24 hours. **B** Cell proliferation was determined by WST-1 proliferation assay (top) and levels of PCNA protein expression by immunoblotting (bottom). Quantification of protein expressions are shown on top. **C** Apoptosis levels as measured by annexin V staining and flow cytometry (top) and genomic DNA fragmentation (bottom). N = 2, data are presented as mean±SE, with triplicate determination. **D** Levels of indicated apoptotic protein by immunoblotting. Quantification of protein expressions are shown on top. **E** Effects of OST-01 on apoptosis of LSC-enriched AML blasts. CD34+CD38- cells were isolated from primary MNCs (n=5) or AML blasts (n=4). Left, levels of DNA fragmentation. Right, levels of PARP and PCNA protein. **F** Effects of OST-01 on colony forming of LSC-enriched AML blasts. HL-60 cells, primary CD34+CD38- AML blasts, or normal mononuclear cells (MNCs) (2 × 10^5^ cells/ml, n=3) were treated with 1 µL/mL of ethanol control or indicated dose of OST-01 for 24 hours before plating on methylcellulose. After 14 days, colonies were images under light microscope (left). Colony number of HL-60 cells, CD34+CD38- AML blasts or MNCs are shown on right and Fig. 1B. N = 2, data are presented as mean±SE, with triplicate determination. Number of colonies are presented as bar graph. Asterisk indicates statistically significant difference based on unpaired t test analysis.

**Figure S2. Effects of OST-01 on USP36-regulated nucleolus structure and ribosome synthesis. A** Table of top 10 up-regulated and down-regulated genes in primary CD34+CD38- AML blasts treated with OST-01 vs vehicle control. The oncogene Myc was among the top five downregulated genes. **B-D** Gene set enrichment analysis (GSEA) graphs of genes involved in Myc targets (**B**), ribosome biogenesis (**C**), ribonucleoprotein complex, ribonucleoprotein complex subunit organization, and ribonucleoprotein complex biogenesis (**D**) upon treatment of primary CD34+CD38- AML blasts with ethanol control or OST-01 (1 µL/mL) for 24 hours. N = 2. ES enrichment score, NES normalized enrichment score, FDR false discovery rate. **E**-**G** HL-60 cells were treated with ethanol control or OST-01 (1 µL/mL) for 24 hours. **E** Effects of OST-01 on expression of deubiquitinase USP36 and nucleoprotein. Immunoblotting of indicated antibodies are shown. Quantification of protein expressions are shown on top. **F** Effects of OST-01 on nucleolus structure. Transmission electron microscope (TEM) was performed to image nucleolus structure. Enlarged images are shown on the right. FC, fibrillary center; DFC, dense fibrillar component; GC, granular component. **G** Effects of OST-01 on number of ribosomes. Left, TEM images of ribosome. Right, quantification of ribosome levels. Asterisk indicates significantly different based on unpaired t test analysis.

**Figure S3. Identification and characterization of principal molecule in OST-01. A** Schematic representation of purification and identification process of active principal molecule from OST-01. **B** HPLC-UV chromatograms of flash chromatography of an active fraction at a wavelength λ226 nm. **C** Bioactivity of active fraction. MV-4-11 cells were treated with ethanol control, a non-active fraction (Non Active F), and dose dependent of an active fraction or OST-01 (1x, 1μl/mL; 2x, 2μl/mL; 3x, 3μl/mL). Apoptosis levels were measured by annexin V staining and flow cytometry. **D** Mass LC-MS/MS spectrum profile of bioactive purified sub-fractions. The molecular masses of peaks are shown. **E** Left, structure of an identified principal molecule, (2E)-21-Hydroxy-2-henicosenoic acid (2E-21). Right, bioactivity of 2E-21 on leukemic cells. Primary CD34+CD38- AML blasts were incubated with indicated doses of 2E-21 (μM) for 24 hours. Apoptosis levels were measured by annexin V staining and flow cytometry. N = 2, data are presented as mean±SE, with triplicate determination. **F** and **G** Effects of 2E-21 on nucleolus structure and number of ribosomes. Primary CD34+CD38- AML blasts were treated with ethanol control or 2E-21 (50 µM) for 24 hours. Transmission electron microscope (TEM) was performed to image nucleolus structure (**F**) and number of ribosomes (**G**). **F** Nucleolus structure. Enlarged images are shown on the right. FC, fibrillary center; DFC, dense fibrillar component; GC, granular component. **G** Number of ribosomes. Left, TEM images of ribosome. Right, quantification of ribosome levels. Asterisk indicates significantly different based on unpaired t test analysis. **H** and **I** Effects of 2E-21 on expression and cellular distribution of nucleolar proteins. Primary CD34+CD38- AML blasts were treated with ethanol control or 2E-21 (50 µL/mL) for 24 hours. **H** The lysates were immunoblotted with indicated antibodies. **I** The cells were stained with indicated antibodies and the images were taken under a confocal microscope. Scale bar, 10 µm.

**Figure S4. Antileukemic activities of OST-01 *in vivo*. A** and **B** Related to Fig. 2A. **A** 0.5x10^6^ luciferase-expressing FLT3-ITD+ Molm-13 AML cells (Luc-Molm-13 cells) were injected into immunodeficient NSG mice through tail vein. On day 7, mice were randomly divided in two groups (each group, n=10) and treated with either OST-01 or vehicle (ethanol) [1 µL/g, oral gavage, BID] continuously until achievement of the euthanized endpoint. Bioluminescence images are presented in Fig. 2A. **B** Kaplan–Meier survival curve of ethanol treated mice (blue line; n = 10; median survival 32 days) or OST-01 treated mice (red line; n = 10; median survival 45.5 days). The statistical significance was determined using Log-rank (Mantel–Cox) test (p < 0.0001). **C-E** Related to Fig. 2B. **C** Experimental design. 1×10^6^ Mll^PTD/WT^/Flt3^ITD/ITD^ bone marrow (BM) MNCs were intravenously injected into sublethally irradiated (4.5 Gy) normal B6 WT recipients. Then, a cohort of mice was divided into 2 groups and treated with either OST-01 (n=10) or vehicle (ethanol, n=8) [1 µL/g/BID, oral gavage]. After 21 days of treatment, the mice were culled to evaluate disease burden, nucleolus structure, ribosome levels, nucleolar protein expression, and perform secondary transplant. Another cohort of mice were followed for survival. **D** Number of LSKs (left) and spleen size (right) are shown. **E** TEM images of ribosome. Other results are presented in Fig. 2B.

**Figure S5. Safety of OST-01 *in vivo-*weight and behavior. A** Toxicology experimental design. Male and female normal black B6 mice (each group, n=5) were treated with 0.5 uL/g, 1.0 uL/g, or 1.5 uL/g of OST-01 or vehicle BID, by oral gavage for 7, 28, or 56 days. **B** The treated mice were observed for weight. No significant changes of weight were observed across all treatment conditions.

**Figure S6. Safety of OST-01 *in vivo-*blood count and pathology analysis.** Male and female normal black B6 mice (each group, n=5) were treated with 0.5 uL/g, 1.0 uL/g, or 1.5 uL/g of OST-01 or vehicle BID, by oral gavage for 7, 28, or 56 days as described in Supplementary Fig. S5A. The treated mice were measured complete blood count (CBCs) (**A**), or tissues (brain, heart, kidney, liver, lung, and spleen) were collected for pathology analysis (**B** and **C**). No significant difference of CBCs and pathology analysis were observed across all treatment conditions.

**Figure S7.**  **Synergistic effects of OST-01 and venetoclax *in vitro* and *in vivo***. **A**-**B** Synergistic effects of OST-01 and venetoclax (VEN) on LSC-enriched AML blasts. Primary CD34+CD38- AML blasts (1 × 10^5^ cells/ml, n=3) were treated with indicated concentration of OST-01 and VEN. Levels of cell proliferation were evaluated (**A**) and synergy score of drug combination was calculated (**B**). Max synergy score: 28.16. **C** Synergistic effects of OST-01 and VEN on apoptosis and colony forming of LSC-enriched AML blasts. Primary CD34+CD38- AML blasts (n=3) were treated with vehicle control, OST-01 (1 µl/mL), VEN (20 nM), or combination of OST-01 and VEN for 24 hours. Left, apoptosis measured by flow cytometry. Right, colony number. **D** OST-01 and VEN combined treatment experimental design. 0.5×10^6^ Mll^PTD/WT^/Flt3^ITD/ITD^ or 1×10^6^ inv(16) BM MNCs were intravenously injected into sublethal irradiated (4.5 Gy) normal B6 WT recipients. The transplanted mice were then randomly divided into 4 groups (n=10-15/group) and treated with either vehicle, OST-01 (1 µL/g/BID, oral gavage, continuously until the euthanized endpoint), VEN (50 mg/kg, once a day, oral gavage, 21 days) or OST-01/VEN at the same doses of single agents. On day 21, 10^6^ BM MNCs cells from each treatment group were harvested for secondary transplant. **E** and **F** Effects of OST-01 on PDX AML model. hCD45+ BM FLT3-WT AML cells (1×10^6^ cells/mouse) were transplanted into NSG mice to generate a cohort of AML bearing PDX mice. The transplanted mice were treated with either vehicle, OST-01 (1 µL/g/BID, oral gavage, continuously until the euthanized endpoint), VEN (50 mg/kg, once a day, oral gavage, 21 days) or OST-01/VEN at the same doses of single agents. **E** Left, Representative images of spleens from recipients of ethanol control, OST-01, VEN, or OST/VEN group. Right, spleen weight from treated recipients of ethanol control (black; 0.39g; n=7), VEN (green; 0.34g; n=7), OST-01 (blue; 0.24g; n=7), or OST/VEN (red; 0.18g; n=7). **F** Left, frequency of hCD33+CD45+ AML cells in the BM of primary transplant mice treated with ethanol control (black; 80.49%; n=7), VEN (green; 64.09%; n=7), OST-01 (blue; 65.23%; n=7), or OST/VEN (red; 37.97%; n=7). Right, frequency of hCD45+ AML cells in the PB of second transplant recipients treated with ethanol control (black; 56.28%; n=8), VEN (green; 51.79%; n=8), OST-01 (blue; 40.23%; n=8), or OST/VEN (red; 14.87%; n=8).
