# Supplementary figures and images for: OST-01, a natural product from Baccharis coridifolia, targets c-Myc-dependent ribogenesis in acute myeloid leukemia
Source: Leukemia. 2024 Jan 17;38(3):657–62. doi: 10.1038/s41375-024-02146-5 (PMC10912030; doi:10.1038/s41375-024-02146-5)

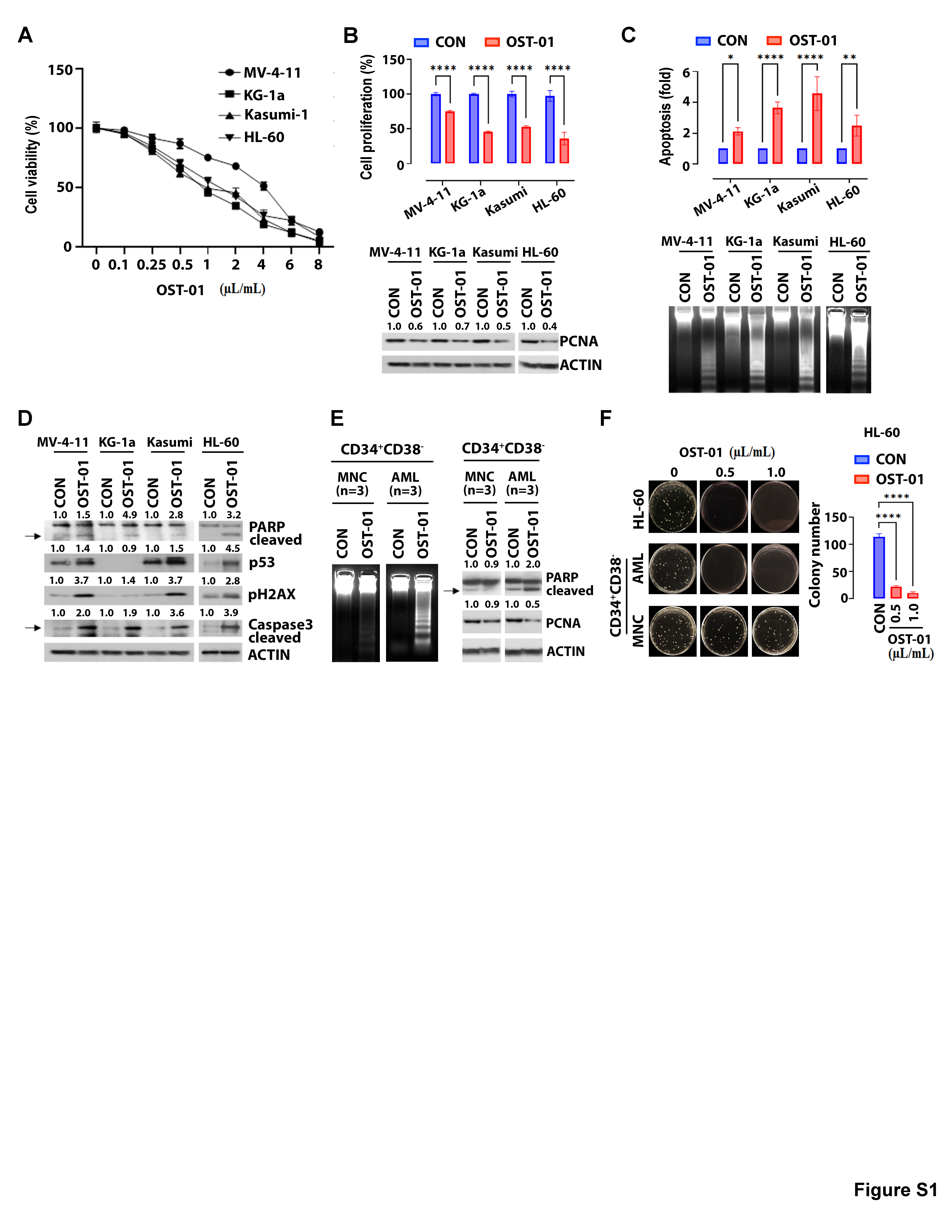

Supplement: Supplementary file 2 — Figure S1 [file 41375_2024_2146_MOESM2_ESM.tif]

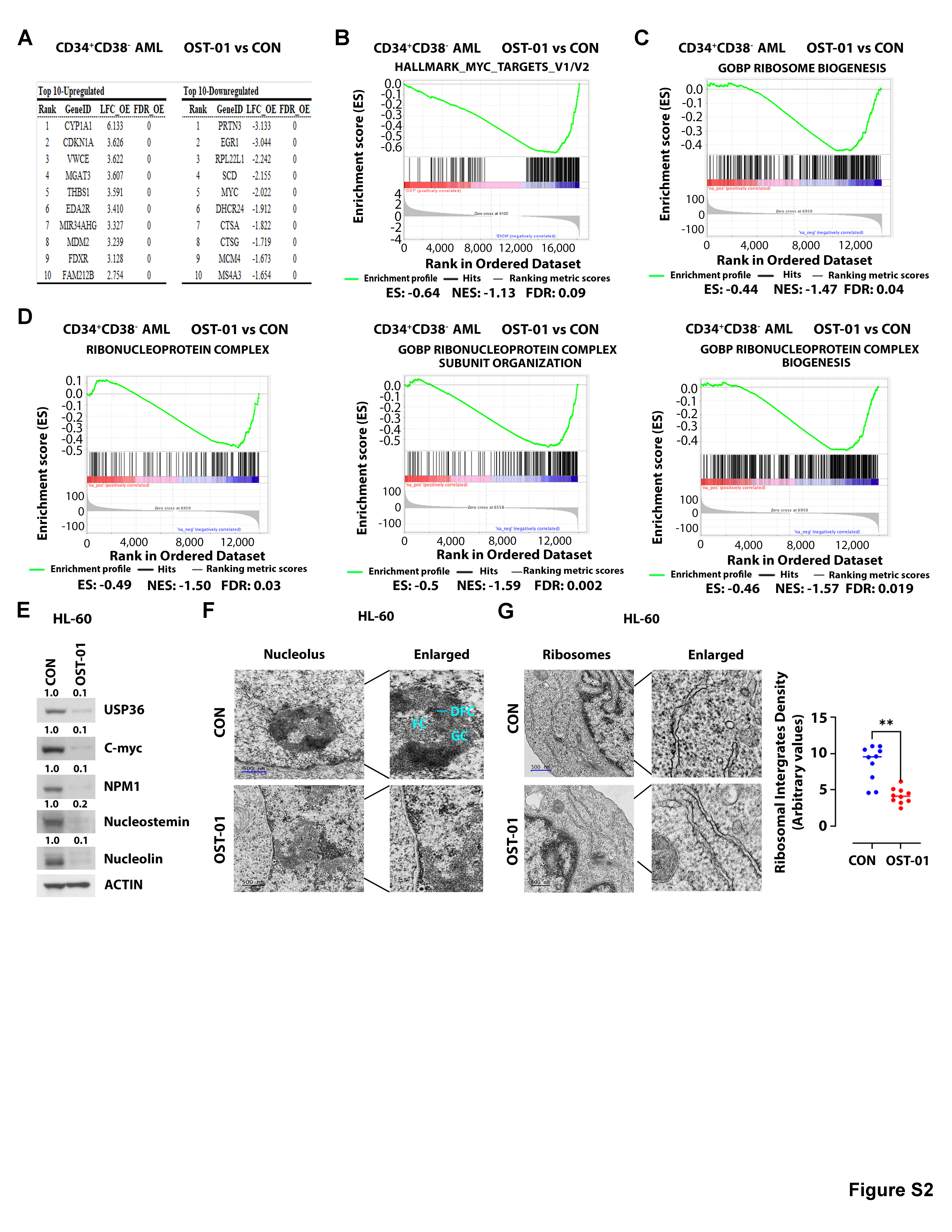

Supplement: Supplementary file 3 — Figure S2 [file 41375_2024_2146_MOESM3_ESM.tif]

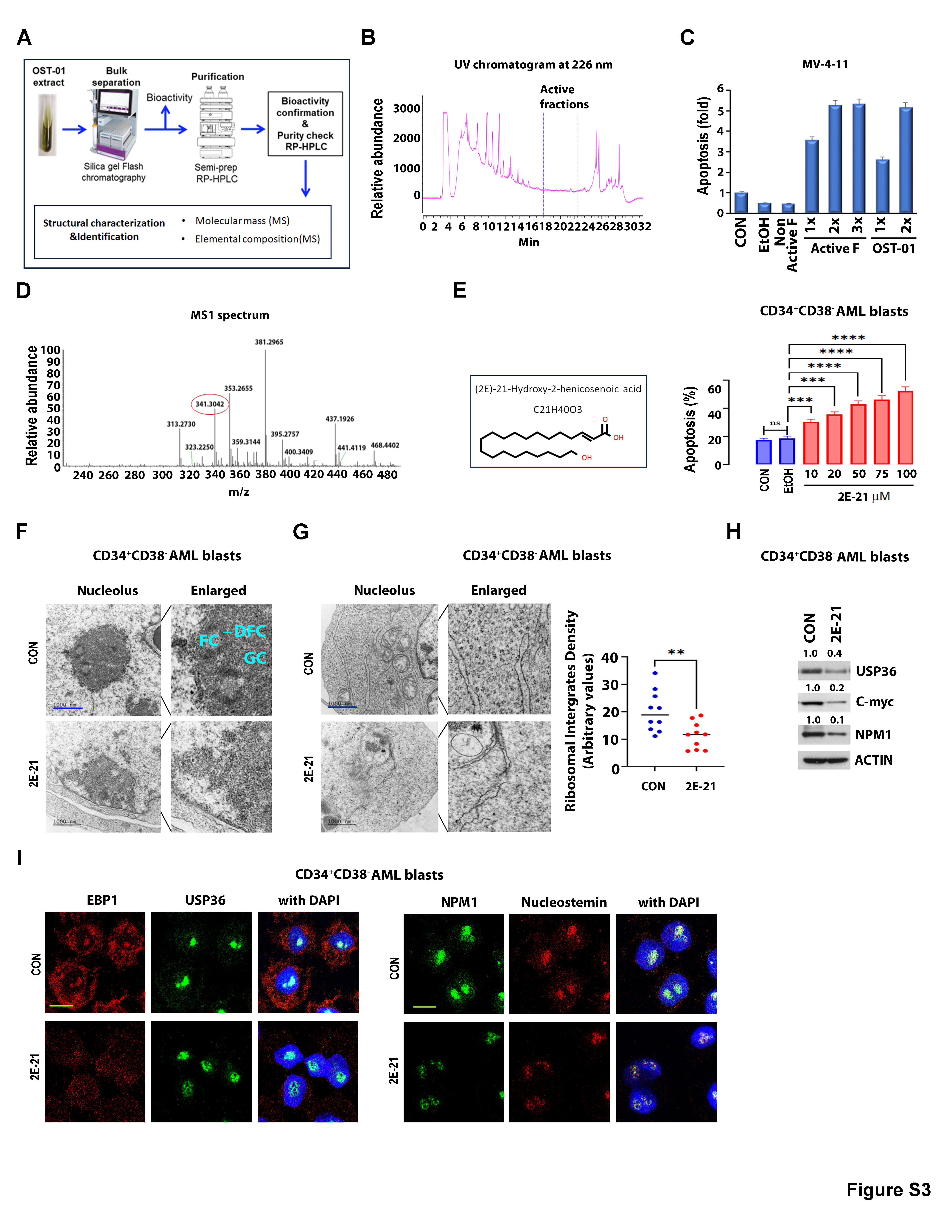

Supplement: Supplementary file 4 — Figure S3 [file 41375_2024_2146_MOESM4_ESM.tif]

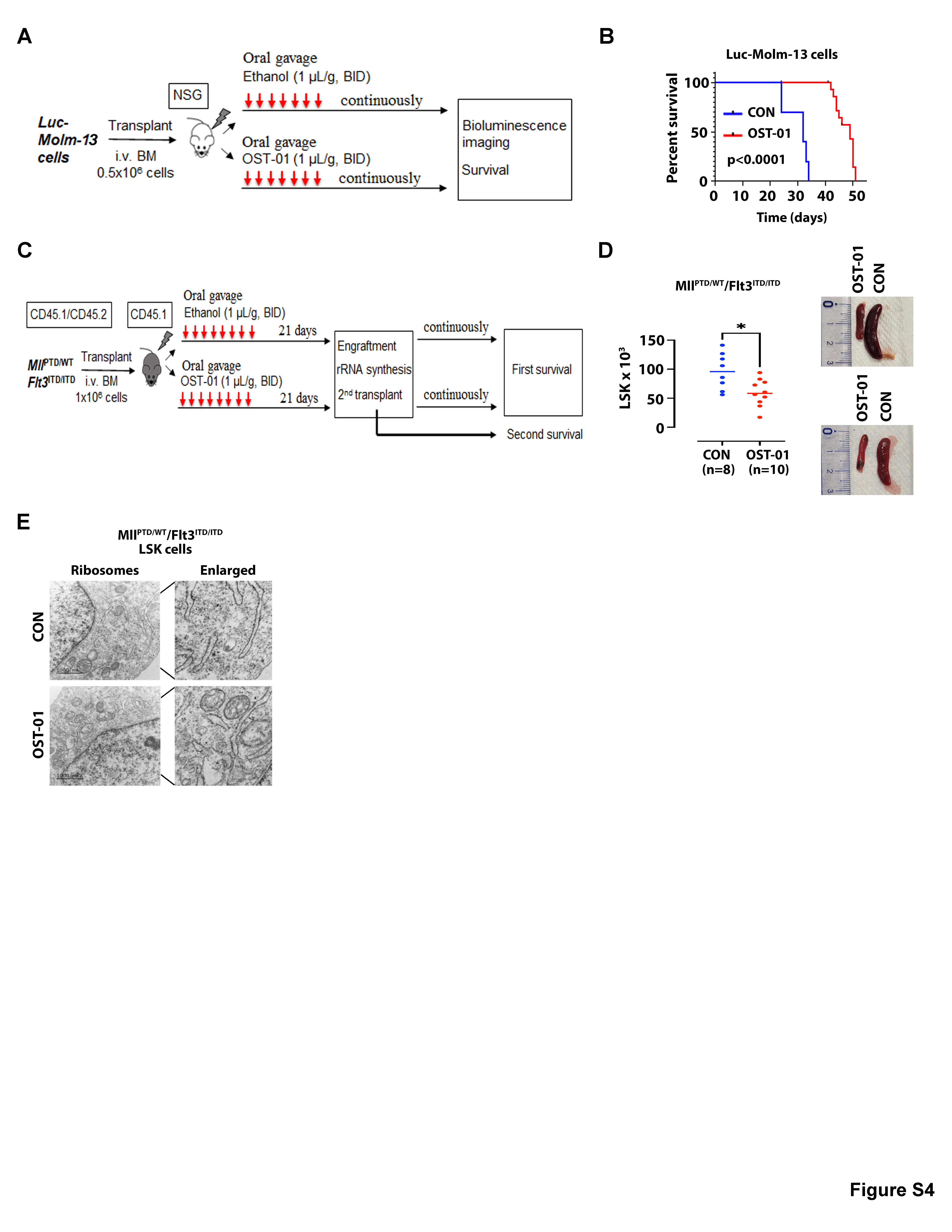

Supplement: Supplementary file 5 — Figure S4 [file 41375_2024_2146_MOESM5_ESM.tif]

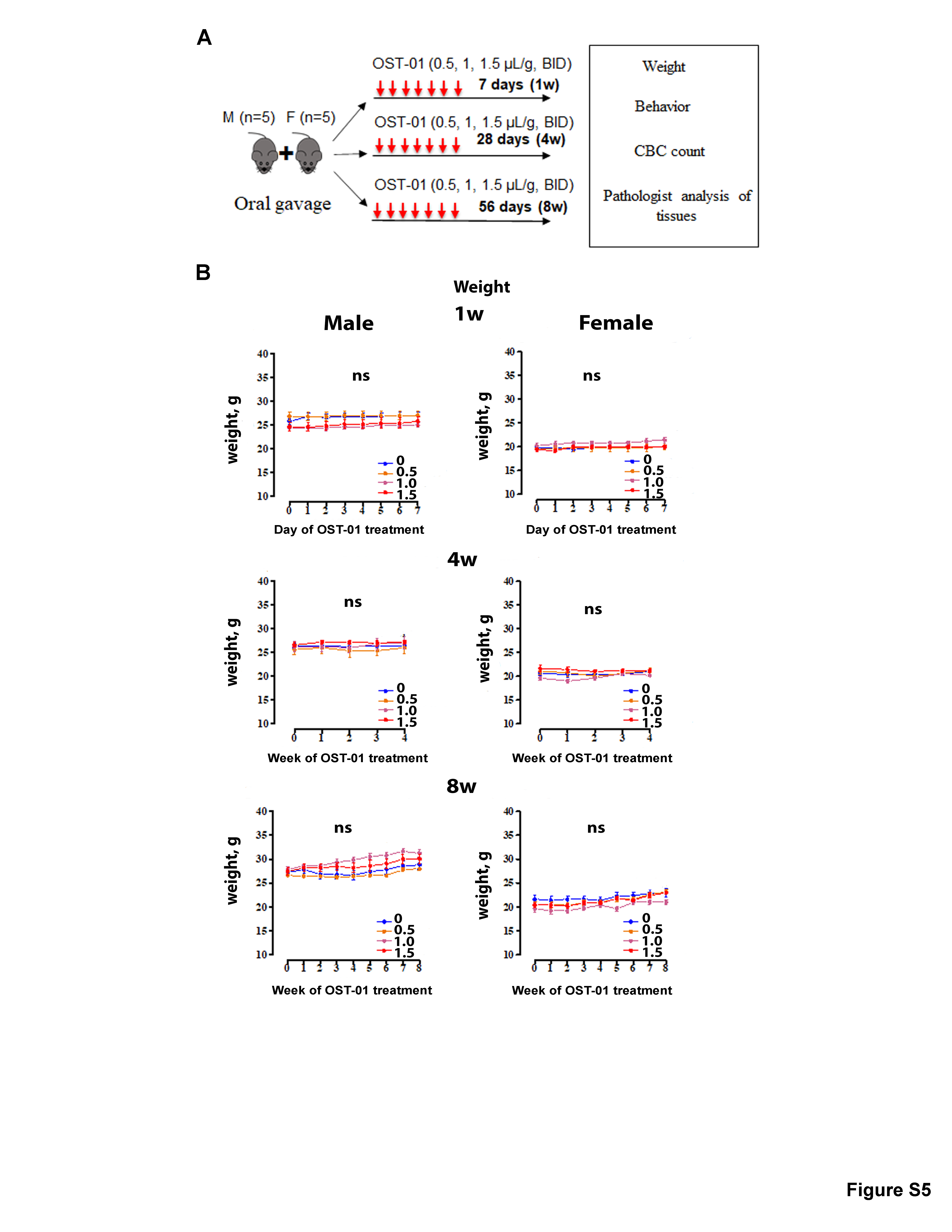

Supplement: Supplementary file 6 — Figure S5 [file 41375_2024_2146_MOESM6_ESM.tif]

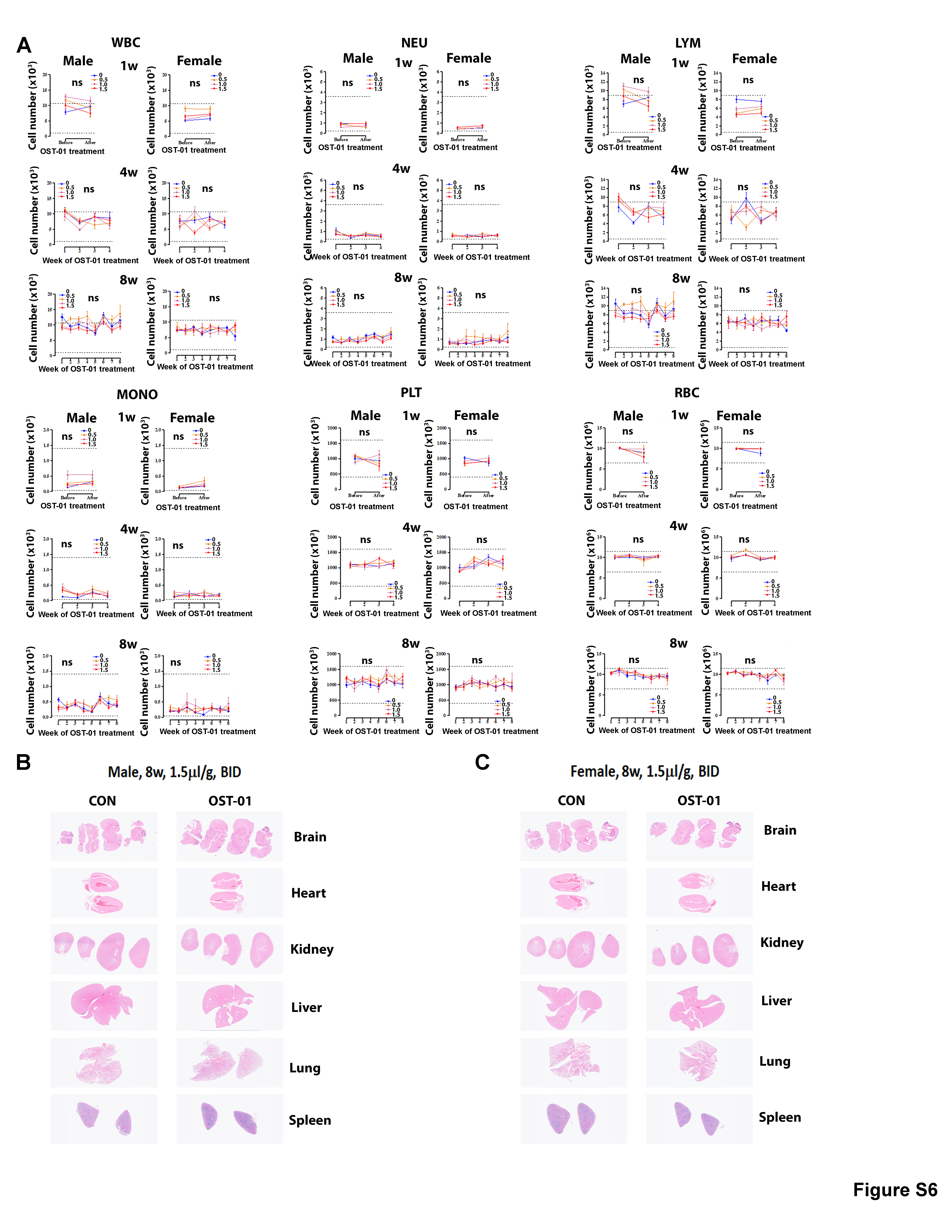

Supplement: Supplementary file 7 — Figure S6 [file 41375_2024_2146_MOESM7_ESM.tif]

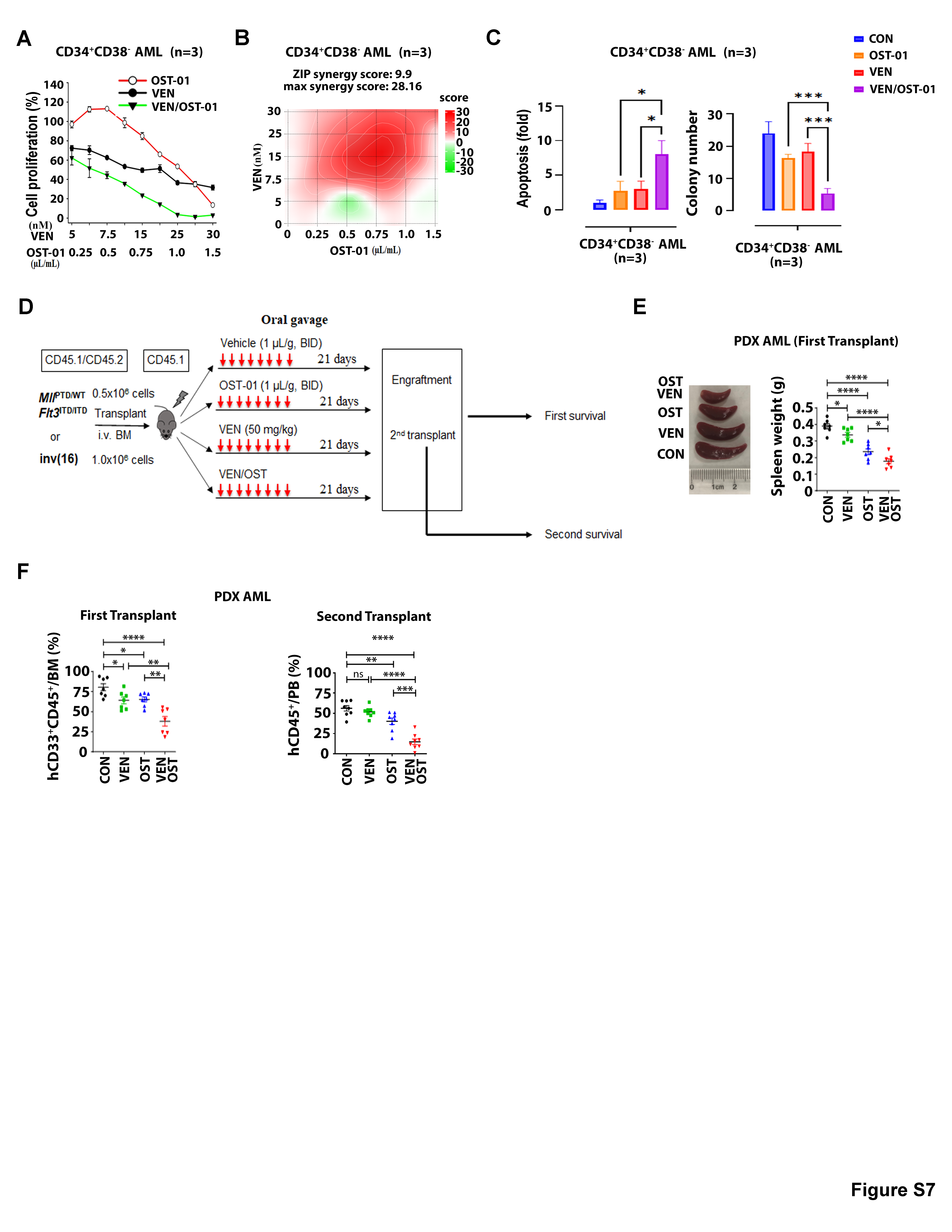

Supplement: Supplementary file 8 — Figure S7 [file 41375_2024_2146_MOESM8_ESM.tif]
